# *Ex vivo* precision-cut liver slices model disease phenotype and monitor therapeutic response for liver monogenic diseases

**DOI:** 10.12688/f1000research.142014.2

**Published:** 2024-04-08

**Authors:** Dany Perocheau, Sonam Gurung, Loukia Touramanidou, Claire Duff, Garima Sharma, Neil Sebire, Patrick F Finn, Alex Cavedon, Summar Siddiqui, Lisa Rice, Paolo G.V. Martini, Andrea Frassetto, Julien Baruteau

**Affiliations:** 1 Great Ormond Street Institute of Child Health, University College London, London, England, WC1N 1EH, UK; 2 Great Ormond Street Hospital for Children NHS Foundation Trust, London, England, WC1N 3JH, UK; 3 Moderna Inc., Cambridge, MA 02139, USA; 4 National Institute of Health Research, Great Ormond Street Biomedical Research Centre, London, WC1N 1EH, UK

**Keywords:** precision-cut tissue slices, vibratome, liver, urea cycle, Argininosuccinic aciduria, Citrullinemia type 1, lipid nanoparticle, mRNA

## Abstract

**Background:**

In academic research and the pharmaceutical industry, *in vitro* cell lines and *in vivo* animal models are considered as gold standards in modelling diseases and assessing therapeutic efficacy. However, both models have intrinsic limitations, whilst the use of precision-cut tissue slices can bridge the gap between these mainstream models. Precision-cut tissue slices combine the advantage of high reproducibility, studying all cell sub-types whilst preserving the tissue matrix and extracellular architecture, thereby closely mimicking a mini-organ. This approach can be used to replicate the biological phenotype of liver monogenic diseases using mouse models.

**Methods:**

Here, we describe an optimised and easy-to-implement protocol for the culture of sections from mouse livers, enabling its use as a reliable *ex-vivo* model to assess the therapeutic screening of inherited metabolic diseases

**Results:**

We show that precision-cut liver sections can be a reliable model for recapitulating the biological phenotype of inherited metabolic diseases, exemplified by common urea cycle defects such as citrullinemia type 1 and argininosuccinic aciduria, caused by argininosuccinic synthase (ASS1) and argininosuccinic lyase (ASL) deficiencies respectively.

**Conclusions:**

Therapeutic response to gene therapy such as messenger RNA replacement delivered via lipid nanoparticles can be monitored, demonstrating that precision-cut liver sections can be used as a preclinical screening tool to assess therapeutic response and toxicity in monogenic liver diseases.

## Introduction

Isolated primary cells and cell line cultures are usually models of choice for *in vitro* studies due to their easy access and low maintenance. However, limitations include the rapid loss of differentiation and lack of a tissue specific microenvironment. ^1^^,^^2^ This can partially be overcome with three-dimensional systems such as spheroids ^3^ and whole-organ bioreactors ^4^; however, these techniques can be technically challenging and costly. Transgenic animals present their own limitations too *i.e.* high maintenance cost and experimental restrictions for ethical reasons. Precision-cut tissue slices (PCTS) fill a gap between such *in vitro* and *in vivo* models and mimic a mini-organ model whilst preserving the tissue architecture and extracellular matrix. ^5^^,^^6^ The development of tissue slicers, *e.g.* vibratomes, ^7^ has allowed the generation of thinner slices with better preserved structural integrity. They can be generated from a wide range of organs, ^8^^–^^11^ tumours ^12^^,^^13^ but also human surgical wastes. ^14^^,^^15^ Also, one organ can generate multiple PCTS, thereby reducing drastically the number of animals but also limiting interindividual variations and off-target effects.

Developing liver PCTS is an appealing strategy to model a disease phenotype such as chronic liver diseases and test preclinical therapeutic effect and potential toxicity.

Here, we present an optimised and easy-to-implement method for the preparation and culture of precision-cut liver slice (PCLS) with survival of up to five days. As an appealing application for a model of chronic liver disease, we show that PCLS recapitulate key phenotypic aspects of two rare inherited metabolic diseases affecting the urea cycle, citrullinemia type 1 and argininosuccinic aciduria (ASA). We also show that PCLS effectively support the proof of concept of non-viral gene therapy by rescuing the ASA phenotype using *hASL* mRNA encapsulated in lipid nanoparticles.

## Methods

### Ethics statement

All animal work was approved following local ethical review by the University College London Animal Welfare and Ethical Review Board and performed under Home Office project license PP9223137 and in accordance with the Home Office (Animals) Scientific Procedures Act (1986). Individual Researchers were performing procedure under personal licences I3906A5FA and I42365670.

All efforts were made to limit harm to animals in accordance to standard practice at the Biological Services Unit at University College London. Animal procedures were performed under the UK Home Office licence PP9223137. Animal procedures were compliant with ARRIVE guidelines and The ARRIVE checklist is available on Open Science Framework, DOI: https://osf.io/vz4jp/. ^16^

### Animals

The transgenic mouse strains were purchased from Jackson Laboratory (Bar Harbor, ME): *Asl ^Neo/Neo^* ( *B6.129S7-Asltm1Brle/J*) and *Ass1* ^*fold*^ ( *B6EiP-Ass1 ^fold^/GrsrJ*). The model for citrullinemia type 1, *Ass ^fold/fold^*, contains a single base pair mutation within the ASS gene, and the model recapitulating Argininosuccinic aciduria (ASA), *Asl ^Neo/Neo^*, contains a neomycin (Neo) cassette inserted into the ASL gene and disrupting its transcription. Mice were mated as heterozygous and a total of six mice per strain were used as parents to generate wild-type and homozygous littermate controls. A total of 15 littermates were used to generate the PCLS. Some of the remaining littermates were used as new breeders. Livers were harvested between the ages of day 12 and 19. All animal work was carried at the Biological Services Unit of University College London. Mice had free access to food and water, housed up to five per cages, in Individually ventilated cages with controlled temperature and humidity conditions and with a 12h light cycle.

### Collection of liver and preparation

**Figure 1.**
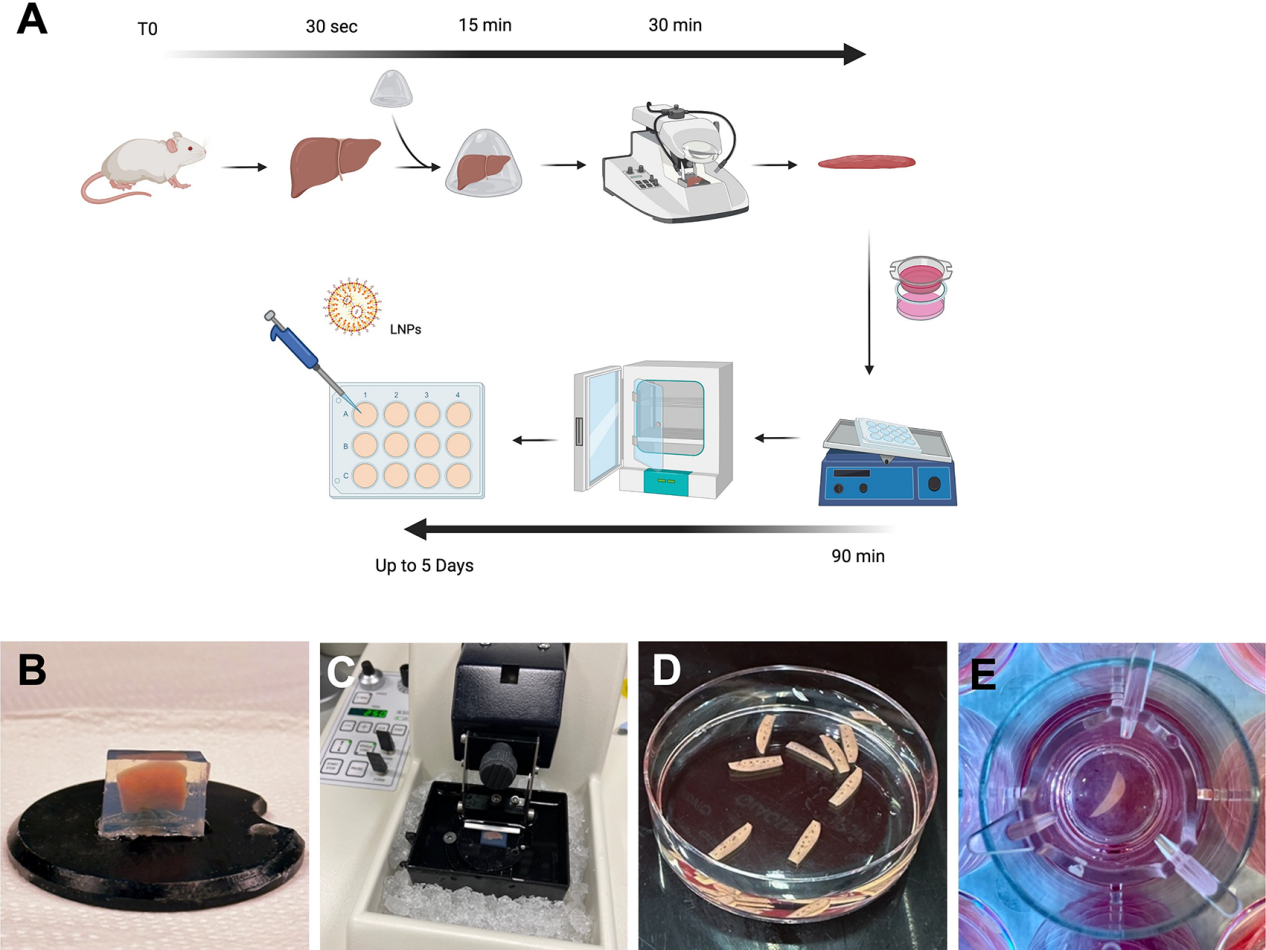
Optimised protocol for cutting and culture of PCLS. (A) Schematic summarising the protocol for generating PCLS. (B) Pre-cut liver lobe embedded in a low-melting agarose block. (C) Agarose block containing the mouse lobe ready for cutting onto vibratome filled with ice and ice-cold Krebs buffer. (D) PCLS following cutting and ready for culture. (E) A PCLS inside a transwell within a 12 well plate containing culture media.

The mouse had been euthanised by raising CO _2_ concentration and according to local procedures. The liver was immediately excised from the mouse and in conditions as sterile as possible and stored directly in ice cold Krebs Buffer. All further steps were performed on ice at 4°C. Each lobe was isolated from the whole liver and all edges further trimmed to obtain a smaller more manageable lobe with straight edges. This helped remove some of the fibrous Glisson’s capsule to further facilitate sectioning. This was performed while keeping the liver surfaces wet into ice cold Krebs buffer ( Figure 1A). One litre of Krebs (Merck, Cat. no K3753) buffer was prepared by dissolving one vial of Krebs powder into 1 L of ultrapure water and kept at 4°C before and during use. Each lobe section was embedded into 4% low melting agarose (ThermoFisher, Cat no 16520050).

### Liver slices preparation

Following harvest, the liver should be processed for cutting as quickly as possible and the time between harvest and culture shouldn’t exceed 3 hours but the shorter this time is, the better with regards to survival. Slicing was performed using a vibratome (Leica, VT1000 S). The agarose blocks were glued, using standard cyanoacrylate glue, directly onto the platform ( Figure 1B) and the tray filled with ice cold Krebs buffer to completely cover the agarose block. The blades (Agar Scientific, Cat no T569T) were placed onto the vibratome at an angle of 10 degree downwards and below horizontal ( Figure 1C). The vibratome was set for cutting at a thickness of 250 μm and speed was set at 5 and frequency at 7. The cutting tray was cooled into the freezer before use and preserved cold with ice around it ( Figure 1C). A spatula was used to collect the liver slices instead of forceps or brushes to avoid damaging the slices.

### Incubation of liver slices

William’s Medium E with GlutaMAX ^TM^ (WME) slice incubation medium was prepared by adding 2 mM L-glutamine supplement (Gibco, Cat no 32551-020), 10% of dialysed FBS (ThermoFisher, Cat no 26400044), 100 U/mL penicillin and 100 μg/mL streptomycin (Gibco, Cat no 15750045), 10 μg/mL Gentamycin (Gibco, Cat no 15750045), 25 mM D-Glucose solution (Gibco, Cat no 15384895), 15 mM HEPES solution (Gibco, Cat no 15630), stored at 4°C. Slices were also cultured using porous 8 μm inserts to allow access to both faces of the slice (Strastedt, Cat no 83.3932.800). Media was also added to the well, enough to slightly cover the slices to create a liquid-air interface while shaking. Plates with slices were transferred into a humidified incubator set to 37°C, 5% carbon dioxide and 20% oxygen level while continuously shaking using an orbital shaker with a speed set at 130 rpm. Thereafter the media was changed every 48 h.

### MTS Assay

The slices were transferred into a 48 well plate containing 400 μl of prewarmed complete WME media and 80 μl of MTS (4,5-dimethylthiazol-2-yl)-5-(3-carboxymethoxyphenyl)-2-(4-sulfophenyl)-2H-tetrazolium) reagent (Abcam, Cat no ab197010) was added. Following incubation for 1 h at 37°C, 5% CO _2_ onto a shaker, 200 μl of media was transferred into a 96 well plate and absorbance was measured at 490 nm. OD raw values were normalised to the fresh weight of individual slices. Slices were weighted at the end of the assay. Individual weights varied from 4.6 mg to 19.1 mg with an average of 10.6 mg.

### Nanoparticle transfection

Codon optimized *hASL* encoding mRNA encapsulated in lipid nanoparticles (LNP-hASL mRNA) were provided by Moderna Therapeutics using their proprietary technology. A total of 2 μg of LNP-hASL in a 10 μL volume were added to the upper side of the slice. This is comparable to an average of 0.19 mg of nanoparticles per gram of liver.

### Citrulline and argininosuccinic acid analysis

Liquid chromatography-Mass spectrometry (LC-MS/MS) was used from dried bloodspots using the hydrophilic interaction liquid chromatography (HILIC) separation of metabolites, method. Briefly, 40 μl of whole blood was spotted on Guthrie blood spot card, dried at room temperature for 24 h and stored in -20°C in a foil bag with desiccant. 3 mm blood spot punch was extracted in 100 μl methanol containing stable isotopes (2 nmol/l, L-citrulline-d7, CDN isotopes, Pomite-Claire, Quebec), used as internal standards, for 15 min in sonicating waterbath at room temperature. The supernatant was collected and dried using Eppendorf ^®^ Concentrator Plus and resuspended in 80 μl of 0.05 M HCl, topped with 280 μl of Solvent A (10 mM ammonium formiate+85% Acetonitrile (ACN)+0.15%Formic acid (FA)), centrifuged at 16,000 rpm for 5 min and supernatant taken for analysis.

Acquity UltraPure Liquid Chromatography (UPLC)-system (Waters, Manchester, UK) using Acquity UPLC BEH Amide column (2.1×100 mm, 1.7 μm particle size) and Van Guard ^TM^ UPLC BEH Amide pre-column (2.1×5 mm, 1.7 μm particle size) (Waters Limited, UK) was used for amino acid chromatography. The mobile phases were (A) 10 mM ammonium formiate in 85% ACN and 0.15% FA and (B) 15 mM ammonium formiate containing 0.15% formic acid, pH 3.0. Detection was performed using a tandem mass spectrometer Xevo TQ-S (Waters, Manchester, UK) using multiple reaction monitoring in positive ion mode. The dwell time was set automatically with MRM-transition of 291.2>70.2, 273.2>70.2 and 176.1>159 respectively for ASA, ASA-anhydrides and L-citrulline. L-Citrulline-d7 (183.15>166.05) was used as internal standard control. Argininosuccinate data were analysed using Masslynx 4.2 software (Micromass UK Ltd, Cheshire, UK).

### ASL enzymatic activity

20-30 mg of liver was homogenised in 400 μl of cold homogenising buffer (50 mM phosphate buffer pH 7.5 and 1x Roche EDTA-free protease inhibitor (Roche, Switzerland)) using Precellys homogeniser tube (VWR, UK) and Precellys 24 tissue homogeniser (Bertin Instruments, France), centrifuged at 10000 g for 20 min at 4°C and protein levels measured from the supernatant using BCA kit (Thermo Fisher Scientific, UK). 60 μg of protein lysate was incubated with 3.6 mM ASA in final volume of 50 μl, incubated at 37°C for 1h followed by reaction termination at 80°C for 20 min. The mixture was centrifuged at 10000 g for 5 min and 5 μl of the supernatant was used to measure fumarate levels per instruction from the commercial fumarate kit (Abcam, Cambridge, UK).

### Statistical analyses

GraphPad Prism 9.0 software (San Diego, CA, USA) was used for performing data analysis and generating graphs. The statistics for this research could be reproduced using the open-source graphical program for statistical analysis JASP.

## Results

### Key aspects of PCLS culture to allow viability over five days

**Figure 2.**
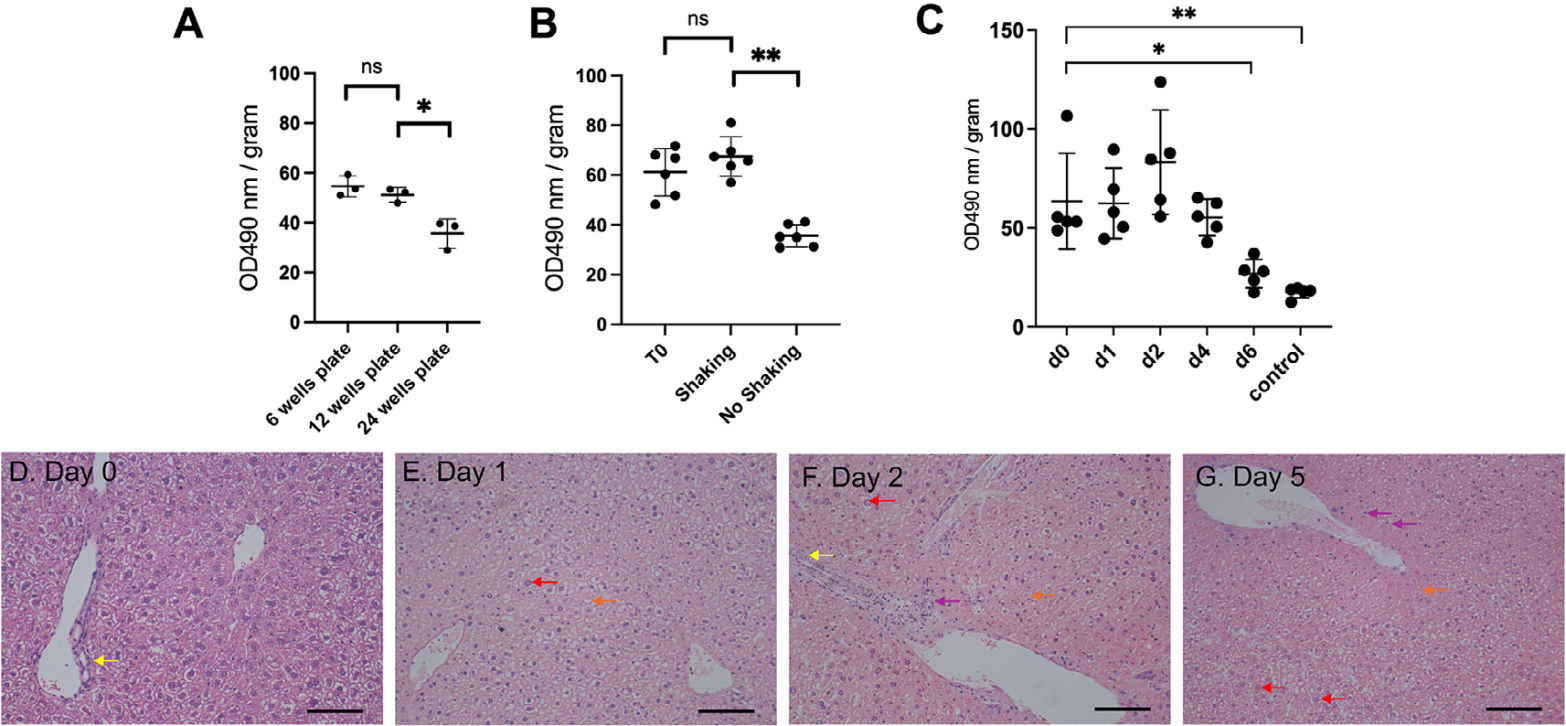
An optimised protocol of PCLS culture shows satisfactory viability for 5 days. (A) Effect of well size on cell viability (n=3). (B) Effect of shaking on cell viability (n=6 per condition). (C) MTS cell viability assay from liver sections from baseline until 6 days of incubation (n=5 per timepoint). OD: arbitrary unit of optical density, normalised to slice fresh weight. Graphs show mean ± SD. Unpaired 2-tailed Student’s t test, ns=not significant, *p<0.05, **p<0.01. (D-G) Representative images of histology of liver PCTS following H&E staining (n=3). Scale bar=100 μM.

Protocols for PCLS preparation and culture vary significantly in the literature with a lack of standardisation, especially for slicing equipment, culture media, and engineering system. Optimisation is always key and varies noticeably depending on the tissue of interest. Here, we recapitulate a rapid and optimised protocol to generate PCLS ( Figure 1). We observed that a minimal volume of culture medium was essential to sustain viability. A reduced volume in 24-well plate showed a significant reduction of viability (p=0.02) compared to 12-well and six-well plates ( Figure 2A, Underlying data ^16^). In agreement with others, ^17^ the use of 12-well plates appears as the best option for optimal survival providing more nutrients and diluting toxic bile acid products. Approximately fifty percent reduction of PCLS viability was also observed without continuous shaking ( Figure 2B, Underlying data ^16^). Shaking creates a critical air-liquid interface, a constant flow, and combined with the use of transwells, increases access to nutrients and oxygen.

With these modifications, viability was assessed and remained constant before observing a significant decrease (p=0.05) at day six ( Figure 2C, Underlying data ^16^). A survival of 5 days refers to an experimental design from day 0 (day of animal harvest, organ slicing and first day of incubation) up to day 4 (5th day of incubation). The PCLS morphology showed no change of bile ducts and architecture up to five days post-incubation ( Figures 2D–G). Only nuclear hyperchromasia, mild inflammatory infiltrates and vacuolisation were observed at day five ( Figure 2G). Taken together, we showed that our optimised PCLS culture protocol enabled viability for five days.

### PCLS recapitulate the disease phenotype of argininosuccinic synthase (ASS) and ASL deficiencies and show proof of concept of mRNA replacement therapy *ex vivo*

**Figure 3.**
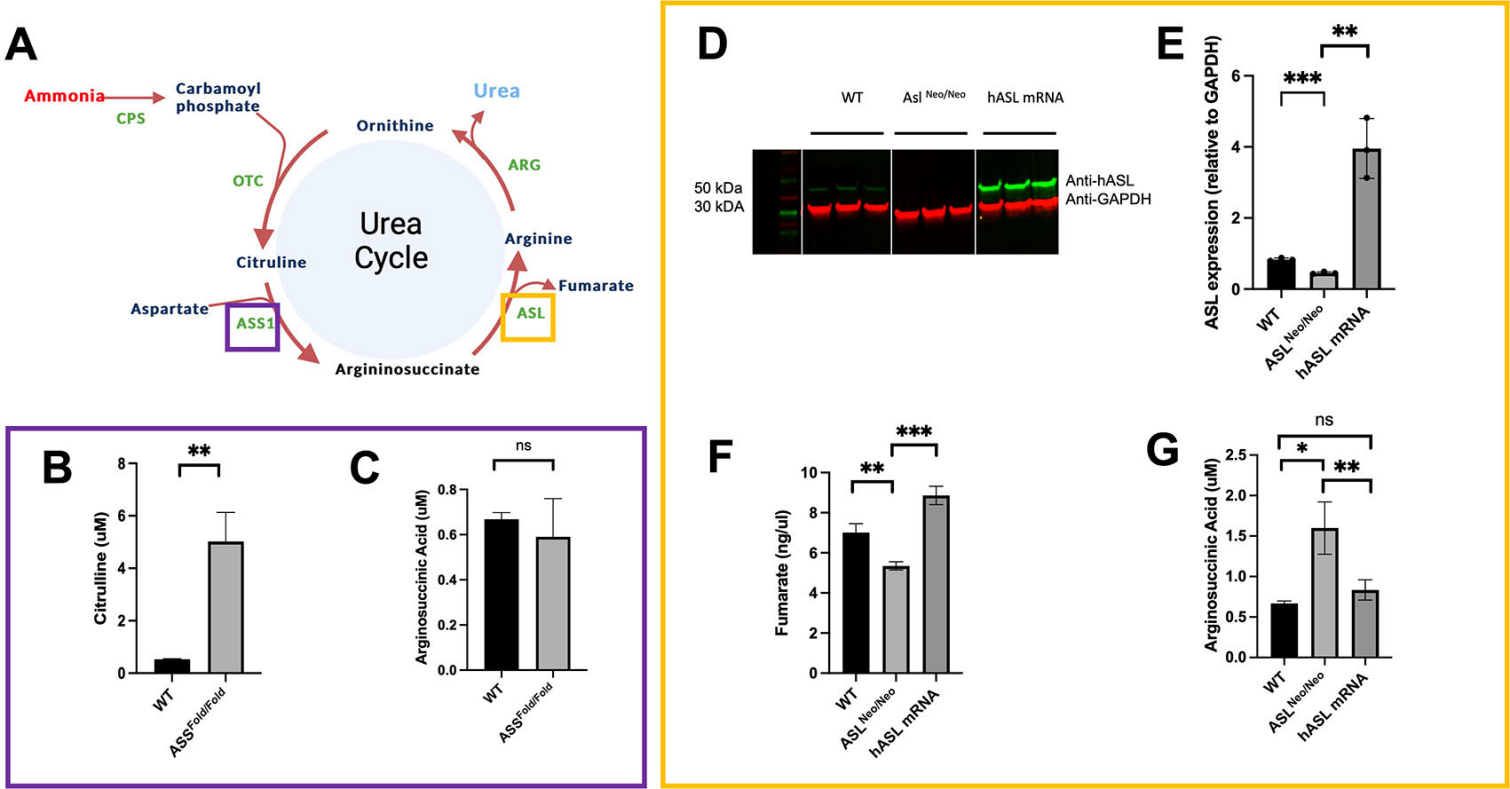
PCLS recapitulate key aspects of the disease phenotype of ASS1 and ASL deficiencies and shows proof of concept of mRNA therapy *ex vivo.* (A) ASL and ASS1 enzymes enable ammonia detoxification in the liver-based urea cycle. (B) Citrulline levels in media after 48 h of incubation in WT and ASS1-deficient PCLS. (C) Arginosuccininic acid levels in media after 48h of incubation in WT and ASS-deficient PCLS. (D) ASL western blot at 48 hours (cropped). (E) Quantification of ASL immunoblot normalised to GAPDH. (F) Liver ASL activity from WT, untreated *ASL* ^*Neo/Neo*^ and hASL mRNA. (G) Arginosuccininic acid levels in media after 48h of incubation in WT and ASS-deficient PCLS. (B-C) n=2 per group; (D-G) n=3 per group. Graphs show mean ± SD. Unpaired 2-tailed Student’s t test, *p<0.05, **p<0.01, ***p<0.005.

Citrullinemia type 1 and ASA are caused by deficiency of the hepatic urea cycle enzymes argininosuccinic synthetase (ASS1) and ASL, respectively ( Figure 3A, Underlying data ^16^). Patients suffering from these urea cycle disorders develop recurrent hyperammonaemia and subsequent neurological symptoms such as developmental delay, coma and death. Despite best-accepted standard of care combining ammonia scavenger drugs and protein-restricted diet, patients present with high rates of mortality and poor quality of life, ^18^ highlighting high unmet needs. The *ex vivo* models rely on induced pluripotent stem cells (iPSC)-derived hepatocytes with a relative inaccuracy in modelling the disease phenotype partially due to sub-optimal differentiation. ^3^ We therefore established a PCLS model using two hypomorphic mouse models: *Ass ^fold/fold^*, for citrullinemia type I and *Asl* *^Neo/Neo^* for ASA. Both models reproduce the clinical phenotype, characterised by impaired growth, abnormal fur, hyperammonaemia and abnormal plasma amino acid profiles. ^19^^,^^20^

As key biomarker for ASS deficiency, citrulline levels showed a significant eight-fold increase in the media of *Ass ^fold/fold^* PCLS compared to that of wild-type littermates ( *p* = 0.01) ( Figure 3B, Underlying data ^16^). No significant difference was observed in argininosuccinate levels ( Figure 3C, Underlying data ^16^). Arginosuccinate levels remain low compare to levels observed in patients, we believe this is due to the high level of dilution using a liver slice with a small superficie and a thickness of only 250 μm within a relatively high volume of culture.

mRNA encapsulated in lipid nanoparticles is an emerging therapeutic strategy for rare liver inherited metabolic diseases ^21^^,^^22^ and PCLS present themselves as an attractive model to assess such therapies. PCLS generated from *Asl ^Neo/Neo^* mice were therefore treated with either *hASL* mRNA or phosphate buffer saline (PBS). We assessed efficacy by testing argininosuccinate levels in PCLS culture media, ASL protein expression and enzymatic function at 48h post-transfection. An eight-fold increase in ASL protein levels was observed in *hASL* mRNA-treated PCLS compared to untreated ( Figures 3D, 3E, Underlying data ^16^). ASL activity, assessed by fumarate production, was also restored in *hASL* mRNA versus PBS-treated PCLS to supraphysiological levels ( Figure 3F, Underlying data ^16^). Argininosuccinate levels were more than two-fold higher in the media of *Asl ^Neo/Neo^* PCLS that of WT controls and were corrected to that of WT levels after *hASL* mRNA incubation ( Figure 3G, Underlying data ^16^). Taken together, these results demonstrate that PCLS culture can replicate the disease phenotype and subsequently be used to assess therapeutic response to gene therapy.

## Discussion

We demonstrate that PCLS can be an appealing *ex vivo* model to assess biological phenotype and therapeutic efficacy, whilst combining the advantage of respecting the complex liver architecture and reducing the use of animals.

Whilst keeping a simple and easy set-up, our setting optimisation emphasizes key aspects to increase PCLS viability, such as volume of media, a dynamic system and agarose embedding for optimal cutting. Such models can be replicated in a standard cell culture laboratory, which has access to an animal facility and a vibratome. This optimisation allowed a viability maintained for 5 days, within the range of 48h to 10 days as previously described. ^23^

Some previously published protocols required complex settings whilst using oxygen concentration at a rate higher than 80% which theoretically should provide longer viability. ^8^ However, such oxygen concentration is likely to generate toxic reactive oxygen species and subsequent antioxidant responses. ^24^ Technical and safety limitations have also made it difficult to use oxygen-enriched media for culturing PCLS. ^24^^,^^25^ Even if there is no consensus on the level of oxygen for culturing PCLS, a comparison between hyperoxic versus physiological models remains difficult.

The PCLS model is a relatively common model but has not been used previously in modelling liver monogenic diseases and assessing therapeutic response to gene therapy. Our work thereby expands the use of this model for these applications and replicates some key characteristics of Citrullinemia type 1 and ASA confirming the use of PCLS as an *ex-vivo* model for preclinical studies. Our approach could benefit other rare or common liver diseases, non-alcoholic fatty liver disease (NAFLD), ^26^ liver cancer ^27^ or even Fah ^-/-^ Rag2 ^-/-^ Il2rg ^-/-^ (FRG) mice with a chimeric humanised liver. ^28^ Whilst human material is difficult to obtain for technical and ethical reasons and additional variability due to genotyping *i.e.* residual activity and subsequent disease severity, quality of hepatocytes and age of patient at collection; makes such FRG mice, repopulated with primary human hepatocytes ideally from a single donor affected by the disease of interest, an ideal application as a preclinical human liver model for application of PCLS modelling. Such a model would also limit the mentioned variability of studying samples from human donors.

The main limitation associated with PCLS is the inability to maintain a sustained model longer than few days. The transduction of the therapeutic agent in inner cell layers of the PCLS has also been questioned. ^23^ Additionally, the need for larger culture volume to increase PCLS viability is a trade-off for high-throughput screening. This variable also adds an important dilution factor and various biomarkers become below the limit of detection and/or quantification, even for accurate and sensitive methods such as tandem mass spectrometry.

Although this warrants further validation, our experience shows that a target engagement with well-selected efficacy endpoints can be reliably tested in thin PCLS, thus enabling supraphysiological correction with potent therapeutic agents.

## Conclusions

To conclude, we present key steps of a PCLS protocol to use as a reliable *ex vivo* model for liver monogenic diseases such as urea cycle defects. We show proof of concept that this model is successful in assessing therapeutic efficacy of gene therapy. We therefore believe this model should become a more recognised tool for preclinical studies in rare and common liver diseases.

## Data Availability

Open Science Framework: Underlying data for ‘Ex vivo precision-cut liver slices model disease phenotype and monitor therapeutic response for liver monogenic diseases’, https://www.doi.org/osf.io/vz4jp. ^16^ This project contains the following underlying data:
• ASA levels in ASL PCLS • ASL Activity • Citrulline and ASA levels in ASS PCLS • ASL Western blot quantification • MTA Assay data • Original Picture Western Blot Open Science Framework: ARRIVE checklist for ‘Ex vivo precision-cut liver slices model disease phenotype and monitor therapeutic response for liver monogenic diseases’, https://www.doi.org/osf.io/vz4jp. ^16^ Data are available under the terms of the Creative Commons Attribution 4.0 International license (CC-BY 4.0)

## References

[1] Dong L Hao H Han W : The role of the microenvironment on the fate of adult stem cells. *Sci. China Life Sci.* 2015;58:639–648. 10.1007/s11427-015-4865-9 25985755

[2] Ferguson LP Diaz E Reya T : The Role of the Microenvironment and Immune System in Regulating Stem Cell Fate in Cancer. *Trends Cancer.* 2021;7:624–634. 10.1016/j.trecan.2020.12.014 33509688 PMC8318571

[3] Duff C Baruteau J : Modelling urea cycle disorders using iPSCs. *NPJ Regen. Med.* 2022;7:56. 10.1038/s41536-022-00252-5 36163209 PMC9513077

[4] Lorvellec M Pellegata AF Maestri A : An in vitro Whole-Organ Liver Engineering for Testing of Genetic Therapies. *iScience.* 2020;23:101808. 10.1016/j.isci.2020.101808 33305175 PMC7708813

[5] Mondonedo JR Bartolak-Suki E Bou Jawde S : A High-Throughput System for Cyclic Stretching of Precision-Cut Lung Slices During Acute Cigarette Smoke Extract Exposure. *Front. Physiol.* 2020;11:566. 10.3389/fphys.2020.00566 32655401 PMC7326018

[6] Viana F O’Kane CM Schroeder GN : Precision-cut lung slices: A powerful ex vivo model to investigate respiratory infectious diseases. *Mol. Microbiol.* 2022;117:578–588. 10.1111/mmi.14817 34570407 PMC9298270

[7] Iulianella A : Cutting Thick Sections Using a Vibratome. *Cold Spring Harb. Protoc.* 2017;2017:pdb.prot094011. 10.1101/pdb.prot094011 28572189

[8] Pearen MA Lim HK Gratte FD : Murine Precision-Cut Liver Slices as an ex vivo Model of Liver Biology. *J. Vis. Exp.* 2020. 10.3791/60992 32225165

[9] De Kanter R Monshouwer M Draaisma AL : Prediction of whole-body metabolic clearance of drugs through the combined use of slices from rat liver, lung, kidney, small intestine and colon. *Xenobiotica.* 2004;34:229–241. 10.1080/004982502000196758 15204696

[10] Nogueira GO Garcez PP Bardy C : Modeling the Human Brain With ex vivo Slices and in vitro Organoids for Translational Neuroscience. *Front. Neurosci.* 2022;16:838594. 10.3389/fnins.2022.838594 35281505 PMC8908416

[11] Klouda T Kim H Kim J : Precision Cut Lung Slices as an Efficient Tool for ex vivo Pulmonary Vessel Structure and Contractility Studies. *J. Vis. Exp.* 2021. 10.3791/62392 34096923

[12] Zimmermann M Armeanu S Smirnow I : Human precision-cut liver tumor slices as a tumor patient-individual predictive test system for oncolytic measles vaccine viruses. *Int. J. Oncol.* 2009;34:1247–1256. 19360338

[13] Philouze P Gauthier A Lauret A : CD44, gamma-H2AX, and p-ATM Expressions in Short-Term ex vivo Culture of Tumour Slices Predict the Treatment Response in Patients with Oral Squamous Cell Carcinoma. *Int. J. Mol. Sci.* 2022;23. 10.3390/ijms23020877 35055060 PMC8775909

[14] Martin C : Human Lung Slices: New Uses for an Old Model. *Am. J. Respir. Cell Mol. Biol.* 2021;65:471–472. 10.1165/rcmb.2021-0268ED 34348085 PMC8641856

[15] Sewald K Danov O : Infection of Human Precision-Cut Lung Slices with the Influenza Virus. *Methods Mol. Biol.* 2022;2506:119–134. 10.1007/978-1-0716-2364-0_9 35771468

[16] Perocheau D : Ex vivo precision-cut liver slices model disease phenotype and monitor therapeutic response for liver monogenic diseases.(Dataset). *Open Science Framework.* 2023. Reference Source 10.12688/f1000research.142014.2 PMC11016166 38618017

[17] Kerkhof EG van de Graaf IA de Jager MH de : Characterization of rat small intestinal and colon precision-cut slices as an in vitro system for drug metabolism and induction studies. *Drug Metab. Dispos.* 2005;33:1613–1620. 10.1124/dmd.105.005686 16051733

[18] Baruteau J Jameson E Morris AA : Expanding the phenotype in argininosuccinic aciduria: need for new therapies. *J. Inherit. Metab. Dis.* 2017;40:357–368. 10.1007/s10545-017-0022-x 28251416 PMC5393288

[19] Perez CJ Jaubert J Guenet JL : Two hypomorphic alleles of mouse Ass1 as a new animal model of citrullinemia type I and other hyperammonemic syndromes. *Am. J. Pathol.* 2010;177:1958–1968. 10.2353/ajpath.2010.100118 20724589 PMC2947290

[20] Erez A Nagamani SC Shchelochkov OA : Requirement of argininosuccinate lyase for systemic nitric oxide production. *Nat. Med.* 2011;17:1619–1626. 10.1038/nm.2544 22081021 PMC3348956

[21] Martini PGV Guey LT : A New Era for Rare Genetic Diseases: Messenger RNA Therapy. *Hum. Gene Ther.* 2019;30:1180–1189. 10.1089/hum.2019.090 31179759

[22] Gurung S Timmermand OV Perocheau P : mRNA therapy restores ureagenesis and corrects glutathione metabolism in argininosuccinic aciduria. *BioRxiv.* 2022. Reference Source 10.1126/scitranslmed.adh1334 PMC7615535 38198573

[23] Dewyse L Reynaert H Grunsven LA van : Best Practices and Progress in Precision-Cut Liver Slice Cultures. *Int. J. Mol. Sci.* 2021;22. 10.3390/ijms22137137 34281187 PMC8267882

[24] t Hart NA Plaats A van der Faber A : Oxygenation during hypothermic rat liver preservation: an in vitro slice study to demonstrate beneficial or toxic oxygenation effects. *Liver Transpl.* 2005;11:1403–1411. 10.1002/lt.20510 16237692

[25] Szalowska E Stoopen G Rijk JC : Effect of oxygen concentration and selected protocol factors on viability and gene expression of mouse liver slices. *Toxicol. In Vitro.* 2013;27:1513–1524. 10.1016/j.tiv.2013.03.007 23531554

[26] Nagarajan P Mahesh Kumar MJ Venkatesan R : Genetically modified mouse models for the study of nonalcoholic fatty liver disease. *World J. Gastroenterol.* 2012;18:1141–1153. 10.3748/wjg.v18.i11.1141 22468076 PMC3309902

[27] Pascale RM Simile MM Peitta G : Experimental Models to Define the Genetic Predisposition to Liver Cancer. *Cancers (Basel).* 2019;11. 10.3390/cancers11101450 31569678 PMC6826893

[28] Cabanes-Creus M Hallwirth CV Westhaus A : Restoring the natural tropism of AAV2 vectors for human liver. *Sci. Transl. Med.* 2020;12. 10.1126/scitranslmed.aba3312 32908003

