# Supplementary material for: Ex vivo precision-cut liver slices model disease phenotype and monitor therapeutic response for liver monogenic diseases
Source: F1000Res. 2024 Apr 8;12:1580. Originally published 2023 Dec 14. [Version 2] doi: 10.12688/f1000research.142014.2 (PMC11016166; doi:10.12688/f1000research.142014.2)
Supplement: Peer review history

# Reviewer report: Reviewer response for version 2

**DOI:** 10.5256/f1000research.164319.r264017

**Published:** 2024-04-13

**Authors:** Vicente Rubio

**Affiliations:** 1 Instituto de Biomedicina de Valencia (IBV-CSIC), Valencia, Select One, 46010, Spain

I am fully satisfied with the changes made and on how the paper reads now.

Is the work clearly and accurately presented and does it cite the current literature?

Yes

If applicable, is the statistical analysis and its interpretation appropriate?

Yes

Are all the source data underlying the results available to ensure full reproducibility?

No source data required

Is the study design appropriate and is the work technically sound?

Yes

Are the conclusions drawn adequately supported by the results?

Yes

Are sufficient details of methods and analysis provided to allow replication by others?

No

Reviewer Expertise:

Urea cycle disorders

I confirm that I have read this submission and believe that I have an appropriate level of expertise to confirm that it is of an acceptable scientific standard.

# Reviewer report: Reviewer response for version 1

**DOI:** 10.5256/f1000research.155506.r232092

**Published:** 2024-01-22

**Authors:** Vicente Rubio

**Affiliations:** 1 Instituto de Biomedicina de Valencia (IBV-CSIC), Valencia, Select One, 46010, Spain

This paper is a technical report on the value on the use of precision-cut liver slices for the study of two urea cycle disorders and for the application of mRNA therapy for one of these two disorders (ASL deficiency).

 The paper is fine but there is a number of technical details that should be given to allow others reproduce the approach as well as for proper description and understanding.

 1. The concept of liver excision requires indication of how was the animal at the time of excision, Presumably the excision was done on anesthetized animals and was followed by death of the animal by exsanguination or by other procedure. Alternatively, the animal could have been killed humanely and the liver excised. In the first case give full detail on the procedure and anesthetic drug. In the second also give killing procedure and the time from death when the liver was excised.

 2. Data are referred to liver weight. Please indicate in the paper when were the  slices weighted and how was the procedure performed to avoid heating them during weighting. In the eventuality that weighting was done at room temperature at the end of the entire procedure, it would be important to indicate this, and if possible please indicate if weight of the slice changes with days of incubation. In any case, it would be good to give, too the average or range of weights of the individual slices

 3. In the figures data are given for days four and six, and since the data at six are inadequate and those at day 4 are found adequate, it is concluded that up to day five is fine. Please give data for day five too, to prove that these data were fine.

 4. I am not a pathologist but from the size of the nuclei in the first slide and in the other slides, I think that the magnification is higher in the first slide (Fig. 2d) than in the other slides (Fig. 2e an subsequent ones). Therefore, please do review and correct the reference horizontal size bars.

 5. On the same question, you mention the preservation of the liver architecture and also mention structural traits like biliary ducts and vacuoles. Please indicate all the traits mentioned using arrows pointing to the structures in the slides.

 6. Also on the same subject, nuclei appear to be within a white non-stained vacuum that may be disrupted cytoplasm or a large aggregate of non-stainable material (glycogen?) Please explain in the figure caption.

 7. Please give the amount of nanoparticles administered per gram of liver.

 8, Please describe the mutations carried by the hypomorphic mice for the two diseases.

 9. Please explain or at least discuss why the ASA levels are so low in the medium for the slices of the ASL deficiency mouse.

 10. Please review the text for correctness as I have found some slips like missing particles or articles.

Is the work clearly and accurately presented and does it cite the current literature?

Yes

If applicable, is the statistical analysis and its interpretation appropriate?

Yes

Are all the source data underlying the results available to ensure full reproducibility?

No source data required

Is the study design appropriate and is the work technically sound?

Yes

Are the conclusions drawn adequately supported by the results?

Yes

Are sufficient details of methods and analysis provided to allow replication by others?

No

Reviewer Expertise:

Urea cycle disorders.

I confirm that I have read this submission and believe that I have an appropriate level of expertise to confirm that it is of an acceptable scientific standard, however I have significant reservations, as outlined above.

# Response

**Published:** 2024-03-26

**Authors:** Dany Perocheau

**Affiliations:** University College London, London, England, UK

We are grateful to the reviewer for his very helpful comments. Please find below specific answers to each of the comments.

*1. The concept of liver excision requires indication of how was the animal at the time of excision, Presumably the excision was done on anesthetized animals and was followed by death of the animal by exsanguination or by other procedure. Alternatively, the animal could have been killed humanely and the liver excised. In the first case give full detail on the procedure and anesthetic drug. In the second also give killing procedure and the time from death when the liver was excised.*

 We thank the reviewer for this comment. The mouse was euthanised by raising CO2 concentration and according to local procedures and the liver quickly excised and store in ice cold Krebs buffer. The liver should then be processed for cutting as quickly as possible and the time between harvest and culture shouldn’t exceed 3 hours but the shorter this time is, the better with regards to survival. We have updated the manuscript accordingly to make details regarding harvest of the liver more informative.

*2. Data are referred to liver weight. Please indicate in the paper when were the  slices weighted and how was the procedure performed to avoid heating them during weighting. In the eventuality that weighting was done at room temperature at the end of the entire procedure, it would be important to indicate this, and if possible please indicate if weight of the slice changes with days of incubation. In any case, it would be good to give, too the average or range of weights of the individual slices.*

 We thank the reviewer for this comment. Indeed, the slices were weighted at the end of each experiment and following the MTS assay. We have updated the manuscript and also added the average range of weights of individual slices.

*3. In the figures data are given for days four and six, and since the data at six are inadequate and those at day 4 are found adequate, it is concluded that up to day five is fine. Please give data for day five too, to prove that these data were fine.*

 We thank the reviewer for this comment. We do not have data for day 5. When we refer to the slices surviving for 5 days, this refers to an experimental design from day 0 (day of animal harvest, organ slicing and first day of incubation) up to day 4 (5th day of incubation).

*4. I am not a pathologist but from the size of the nuclei in the first slide and in the other slides, I think that the magnification is higher in the first slide (Fig. 2d) than in the other slides (Fig. 2e an subsequent ones). Therefore, please do review and correct the reference horizontal size bars.*

 We thank the reviewer for this comment. We have checked the slices at day 0 and took new pictures and updated the horizontal size bar. The nuclei still appears relatively slightly large on average. We believe this could be an artefact following tissue fixation or staining.

*5. On the same question, you mention the preservation of the liver architecture and also mention structural traits like biliary ducts and vacuoles. Please indicate all the traits mentioned using arrows pointing to the structures in the slides.*

 We thank the reviewer for this comment. We have taken new pictures and more indicated in a clear manner with arrows as suggested the traits mentioned in figure 2.

*6. Also on the same subject, nuclei appear to be within a white non-stained vacuum that may be disrupted cytoplasm or a large aggregate of non-stainable material (glycogen?) Please explain in the figure caption.*

 We thank the reviewer for this comment. We have checked with our pathology department, and we believe this is an artefact due to fixation and processing.

*7. Please give the amount of nanoparticles administered per gram of liver.*

 We thank the reviewer for this comment. The amount of LNP per gram of liver averages to 0.19mg of LNP per gram of liver. We have updated the manuscript accordingly.

*8, Please describe the mutations carried by the hypomorphic mice for the two diseases.*

 We thank the reviewer for this comment. The model for citrullinemia type 1, *Ass ^fold/fold^*, contains a single base pair mutation within the ASS gene, and the model recapitulating Argininosuccinic aciduria (ASA), *Asl ^Neo/Neo^*, contains a neomycin (Neo) cassette inserted into the ASL gene and disrupting its transcription. We have amended the manuscript accordingly.

*9. Please explain or at least discuss why the ASA levels are so low in the medium for the slices of the ASL deficiency mouse.* We thank the reviewer for this comment.  The levels of ASA does appear quite low compare to levels observed in patients. Based on the low thickness of 250 mm and the small superficie of about 0.14 cm ^2 ^within a relatively high volume of 700 ml, the rate of dilution for biomarkers such as ASA becomes quite high which explains why their levels seem low compare to patients for instance. We have amended the results section.

*10. Please review the text for correctness as I have found some slips like missing particles or articles.*

 We thank the reviewer for his comment. We have reviewed the manuscript and corrected it accordingly.

# Reviewer report: Reviewer response for version 1

**DOI:** 10.5256/f1000research.155506.r232090

**Published:** 2024-01-22

**Authors:** Tamir Rashid

**Affiliations:** 1 Imperial College London, London, England, UK

The authors describe an optimised and easy-to-implement protocol for the culture of precision-cut tissue slices (PCTS) from mouse liver, enabling its use as a reliable *ex-vivo* model to assess the therapeutic screening of inherited metabolic diseases.

 They show that PCTS can be a reliable model for recapitulating some key aspects of the phenotype of two inherited metabolic diseases - citrullinemia type 1 and argininosuccinic aciduria, caused by argininosuccinic synthase (ASS1) and argininosuccinic lyase (ASL) deficiencies respectively.

 They also provide *ex vivo *proof of concept data for the use of mRNA therapy in this context.

 Overall this is a simple, clear and helpful piece of work for people working in the field and expands the literature on PCTS.

Is the work clearly and accurately presented and does it cite the current literature?

Yes

If applicable, is the statistical analysis and its interpretation appropriate?

Yes

Are all the source data underlying the results available to ensure full reproducibility?

Yes

Is the study design appropriate and is the work technically sound?

Yes

Are the conclusions drawn adequately supported by the results?

Yes

Are sufficient details of methods and analysis provided to allow replication by others?

Yes

Reviewer Expertise:

Genetic liver diseases; disease modelling

I confirm that I have read this submission and believe that I have an appropriate level of expertise to confirm that it is of an acceptable scientific standard.

# Response

**Published:** 2024-03-26

**Authors:** Dany Perocheau

**Affiliations:** University College London, London, England, UK

We would like to thank the reviewer for the comments and for his time.

 Best wishes

 Dany Perocheau and co authors
